# *Cinnamomum zeylanicum*, *Origanum vulgare*, and *Thymus vulgaris* Essential Oils in Cystitis: An Alternative/Complementary Perspective

**DOI:** 10.3390/antibiotics15090909

**Published:** 2026-09-15

**Authors:** Laura Beatrice Mattioli, Ivan Corazza, Alberto Santini, Carla Marzetti, Maria Frosini, Emanuele Carosati, Diana L. Prado-Romero, Giovanni Scala, Roberta Budriesi, Luca Camarda

**Affiliations:** 1 Department of Pharmacy and Biotechnology, Food Chemistry and Nutraceutical Lab, Alma Mater Studiorum-University of Bologna, 40126 Bologna, Italy; laurabeatrice.mattioli@unibo.it (L.B.M.); l.camarda@unibo.it (L.C.); 2 Interdepartmental Centre for Industrial Research in Health Sciences and Technologies–CIRI Health Sciences and Technologies, Alma Mater Studiorum-University of Bologna, 40138 Bologna, Italy; ivan.corazza@unibo.it; 3 Department of Medical and Surgical Sciences (DIMEC), Alma Mater Studiorum-University of Bologna, 40138 Bologna, Italy; 4 Valsambro S.r.l., via Cairoli 2, 40121 Bologna, Italy; alberto.santini@valsambro.it (A.S.); carla.marzetti@valsambro.it (C.M.); 5 Department of Life Sciences, University of Siena, 53100 Siena, Italy; maria.frosini@unisi.it; 6 Department of Chemical and Pharmaceutical Sciences, University of Trieste, 34127 Trieste, Italy; emanuele.carosati@units.it (E.C.); dianalorena.pradoromero@units.it (D.L.P.-R.); 7 Biology Department, University of Naples “Federico II”, 80138 Naples, Italy; giovanni.scala@unina.it

**Keywords:** antimicrobial activity, cystitis, spontaneous and induced bladder contractility, antioxidant activity, BioGPS, ligand–target complementarity prediction

## Abstract

**Background/Objectives**: Cystitis is a common lower urinary tract infection often associated with recurrence, bladder discomfort, and increasing antimicrobial resistance. Essential oils (*Cinnamomum zeylanicum*, *Origanum vulgare*, and *Thymus vulgaris*) are phytocomplexes with antimicrobial, antioxidant, and smooth muscle-modulating properties. **Methods**: The essential oils (contained in the solid formulations) were tested against 30 samples obtained either from urine cultures or vaginal swabs, which showed either *Escherichia coli* or *Enterococcus faecalis*. Antibacterial activity was assessed by disk diffusion assay: the efficacy of ciprofloxacin was measured, as well as the efficacy of each oil, tested both alone and in combination with ciprofloxacin. Their effects on bladder function were evaluated ex vivo in isolated guinea pig bladder strips by analyzing spontaneous and KCl-induced contractility. Antioxidant activity was determined using the DPPH assay. **Results**: All three essential oils showed antibacterial activity against the tested clinical isolates, with different susceptibility profiles. Their combination with ciprofloxacin generally improved antibacterial responsiveness, including in isolates with reduced ciprofloxacin sensitivity. *O. vulgare* and *T. vulgaris* essential oils showed relevant spasmolytic activity against KCl-induced contraction, whereas *C. zeylanicum* displayed the strongest antioxidant activity and favorable modulation of spontaneous bladder contractility at low concentrations. A combined chemoinformatic and bioinformatic approach enabled the identification of potential interactions between multiple essential oil constituents and the biological pathways that ciprofloxacin is likely to target. **Conclusions**: These findings suggest that cinnamon, oregano and thyme essential oils may act as multitarget phytocomplexes with antibacterial, antioxidant and bladder-modulating properties. They may represent complementary candidates for cystitis management, although further mechanistic, formulation and safety studies are required.

## 1. Introduction

Urinary tract infections (UTIs) are acute inflammatory conditions caused by pathogen proliferation in an otherwise sterile urinary tract [1]; globally, UTIs affect approximately 150 million people each year [2].

These infections are classified by anatomical site: pyelonephritis affects the upper urinary tract and kidneys, whereas lower UTIs include cystitis (bladder), urethritis (urethra), and prostatitis (prostate). Infections can spread between regions, and UTIs often recur [3]. UTIs are considered complicated when they occur in children, pregnant women, or individuals with structural or functional urinary abnormalities, urinary obstruction, or comorbidities [1].

Cystitis, the most common lower UTI, is predominantly bacterial in origin [4]. It can be asymptomatic (asymptomatic bacteriuria) or symptomatic, with dysuria, urinary urgency and frequency, hematuria, cloudy or malodorous urine, and impaired bladder emptying. The urinary tract is normally sterile, and micturition is a key defense against bacterial colonization. Factors such as urinary stasis or retrograde urine flow increase susceptibility to bacterial or fungal infection. In adults aged 20–50, women are more frequently affected due to a shorter urethra, whereas in men, infections are often associated with intravesical devices [5] or benign prostatic hyperplasia [6].

The lower urinary tract is protected by multiple defense mechanisms, including complete bladder emptying, urine acidity, vesicourethral valve function, and mucosal and immunological barriers. The primary pathogens of cystitis are aerobic Gram-negative bacteria, especially *Escherichia coli*, which account for 75–95% of cases [7]. Other occasional agents include *Enterococcus faecalis*, *Klebsiella*, *Proteus*, *Staphylococcus epidermidis*, and *Candida albicans*. Standard treatment involves targeted antibiotics guided by antibiograms or broad-spectrum antibiotics, which may contribute to antimicrobial resistance [8,9].

Adjunctive measures include hydration, dietary management, and antispasmodics to improve bladder function [10], since infection can stiffen bladder tissue and reduce contractility [11].

Biological approaches aimed at modulating the urinary microbiome are also emerging [12]. In addition to pharmacological interventions, dietary factors play a critical role in the prevention and management of UTIs. Adequate fluid intake supports regular micturition, which mechanically reduces bacterial colonization. At the same time, specific dietary components, such as cranberries, probiotics, and vitamin C, can modulate urinary pH, inhibit bacterial adhesion, and enhance host immune defenses [13]. Beyond nutrition, bioactive plant-derived compound mixtures, including essential oils (EOs), offer complementary strategies owing to their antibacterial, anti-inflammatory, and antioxidant properties [14]. EOs, in fact, have demonstrated antibacterial activity both as phytocomplexes and as individual constituents [15]. In addition to these characteristics, EOs counteract biofilms [16] and, in some cases, through the formulation used [17], and may enhance the effectiveness of conventional therapies, supporting the rationale of the study.

Integrating dietary measures with natural products, such as essential oils, may enhance infection control, support tissue health, and reduce reliance on antibiotics, thereby addressing both symptom management and antimicrobial resistance.

Moreover, given the global challenge of antimicrobial resistance [10], exploring alternative or complementary therapies is critical. Previous analyses using bio- and chemoinformatic simulations have led us to hypothesize that EOs from thyme, oregano, and cinnamon can modulate bacterial metabolic and nucleic acid pathways, suggesting potential to enhance antibiotic efficacy [18].

In addition, to verify whether the essential oils under study extend their action to additional nodes of the target network linked to bacterial cystitis, we used a well-established multicomponent multitarget ligand-based approach previously described [19]. We extended the study by investigating the antimicrobial and antioxidant activity of EOs from *Cinnamomum zeylanicum* Ness bark (**CZEO**)*, Origanum vulgare* L. (**OVEO**) flowering tops, and *Thymus vulgaris* L. (**TVEO**) flowering tops. Their effects on bladder function were also explored by evaluating spontaneous and KCl-induced contractility in isolated guinea pig bladder. In addition, their ability to modulate key nodes within ciprofloxacin/cystitis-related target networks is discussed in relation to their chemical composition, providing insight into their potential as complementary or alternative interventions for cystitis management while preliminarily addressing clinical safety and functional impact on the urinary tract.

## 2. Results

In this study, three essential oils (**CZEO**, **OVEO**, and **TVEO**) were evaluated against 30 clinical strains of *Escherichia coli* and *Enterococcus faecalis*, obtained from 19 urine cultures and 11 vaginal swabs, with **CIP** used as a positive control. In parallel, the effects of the essential oils and **CIP** were investigated on representative strains of the urogenital microbiota. In addition, the effects of essential oils on spontaneous and induced contractility of bladder smooth muscle were evaluated.

### 2.1. Determination of Antibacterial Activity

**Figure 1:**
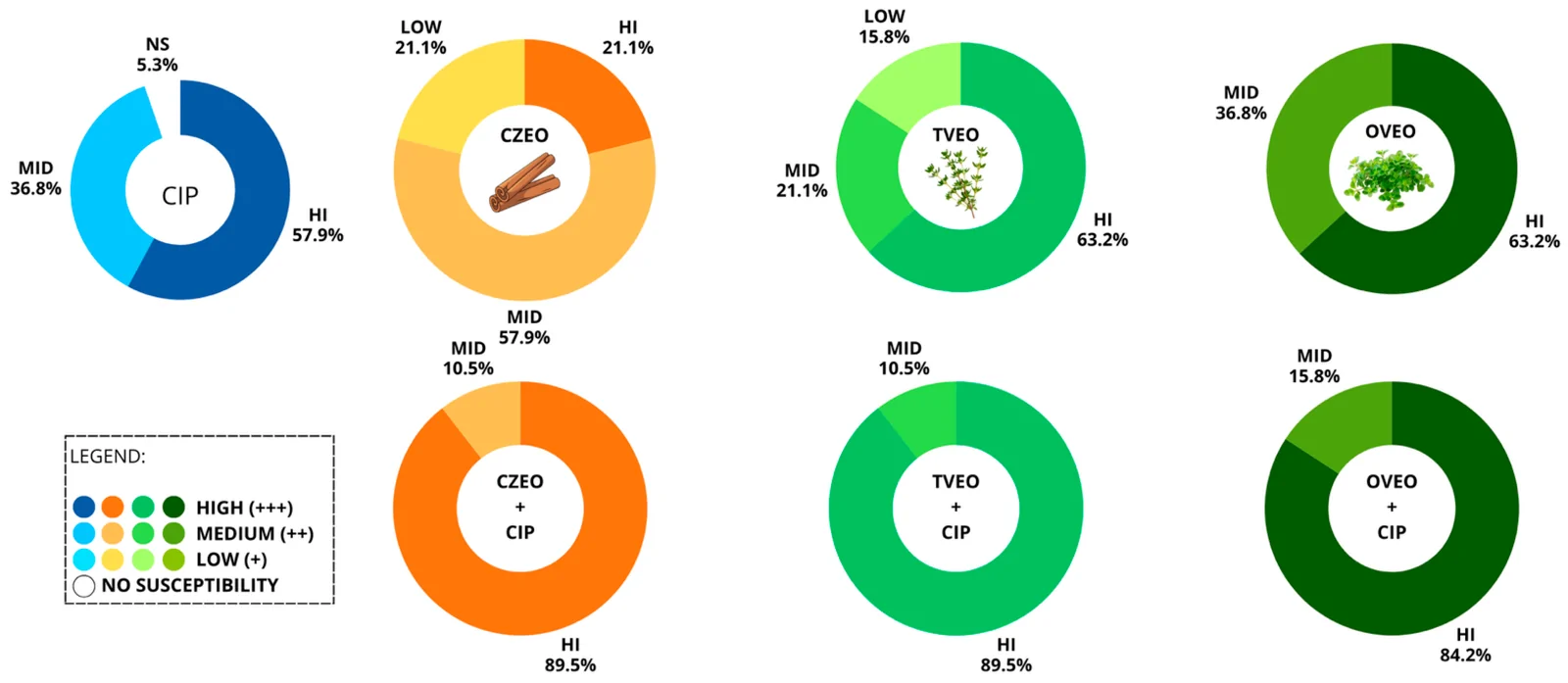
Configuration of the antibacterial activity profile of bacteria obtained from urine cultures.

**Figure 2:**
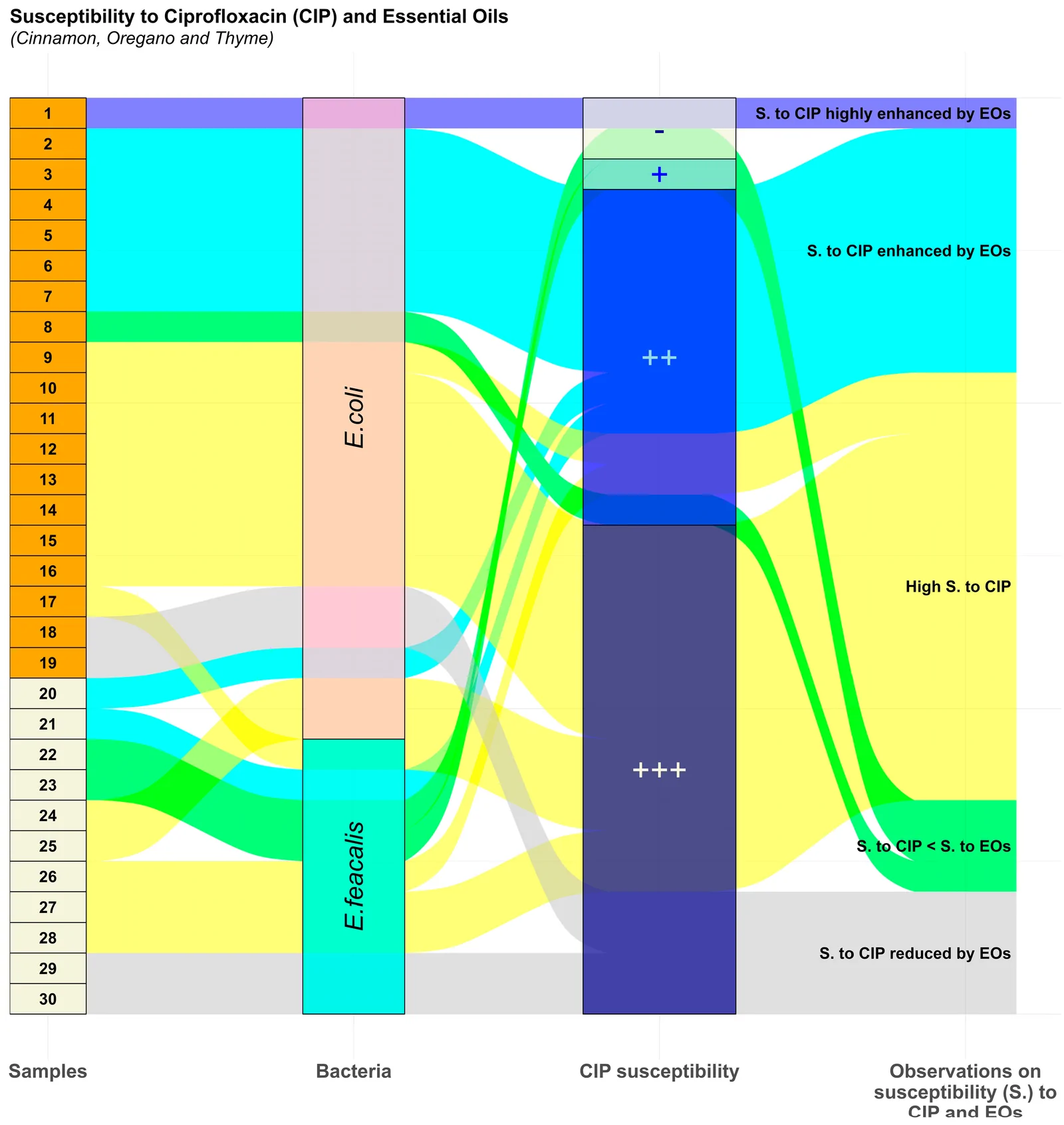
Alluvial plot summarizing the relationships among bacterial isolates, susceptibility to the combination of ciprofloxacin (**CIP**) and essential oils (EOs), and the overall interpretation of the interaction between **CIP** and EOs. Each flow connects individual samples to the identified bacterial species, the degree of susceptibility to **CIP** (−, +, ++, +++), and the final classification of the interaction outcome, including S. to CIP enhanced by EOs, S. to CIP highly enhanced by EOs, S. to CIP reduced by EOs, high S. to **CIP**, or cases in which S. to CIP < S. to EOs (“S.” means susceptibility). The overall interpretation was assigned considering the combined behavior of cinnamon (**CZEO**), oregano (**OVEO**), and thyme (**TVEO**) essential oils.

*Urine cultures.* Data on microorganism identification, susceptibility to CIP (antibiogram), minimum inhibitory concentration (MIC), essential oil (EO) susceptibility (aromatogram), and the combined effects of essential oils and antibiotics for the 19 urine culture samples included in the study are summarized in Table 1. Figure 1 presents the results for *E. coli*. Figure 2 helps the reader follow how different samples are susceptible to EOs and **CIP**, with a focus on the impact of CIP-EOs combinations.

In 95% of the tested urine culture samples, *E. coli* was identified, whereas *E. faecalis* accounted for the remaining 5%.

The antibiogram showed that 95% of the isolates were susceptible to ciprofloxacin, with varying degrees of sensitivity: 58% were highly susceptible (+++, yellow flow; Figure 2), while 37% showed intermediate susceptibility (++).

The aromatograms revealed differential effects, depending on the essential oil tested. Specifically, 100% of the isolates were susceptible to **CZEO**, though to varying degrees: 21.1% showed low susceptibility, 57.9% intermediate susceptibility, and 21.1% high susceptibility. Similarly, 100% of the isolates were susceptible to **TVEO**, with 63.2% classified as highly susceptible, 21.1% as intermediately susceptible, and 15.8% as low susceptible. Regarding **OVEO**, all tested isolates (100%) were susceptible, of which 63.2% were highly susceptible and 36.8% intermediately susceptible.

The combined activity of **CIP** (5 μg) with each essential oil was evaluated across all isolates using 1.10 mg for **CZEO**, 1.20 mg for **OVEO**, and 0.95 mg for **TVEO**. Overall, the combinations resulted in 100% susceptibility among the tested isolates. Specifically, the **CZEO**-**CIP** combination produced 89.5% highly susceptible isolates and 10.5% intermediately susceptible isolates. The TVEO-CIP combination showed an identical distribution (89.5% highly susceptible; 10.5% intermediately susceptible). In the OVE-CIP combination, 84.2% of isolates were highly susceptible, and 15.8% were intermediately susceptible.

Notably, the schematic representation considered the overall effect of the three EOs; in cases of discordant responses among the oils, the classification was assigned according to the response observed for most of the EOs.

**Figure 3:**
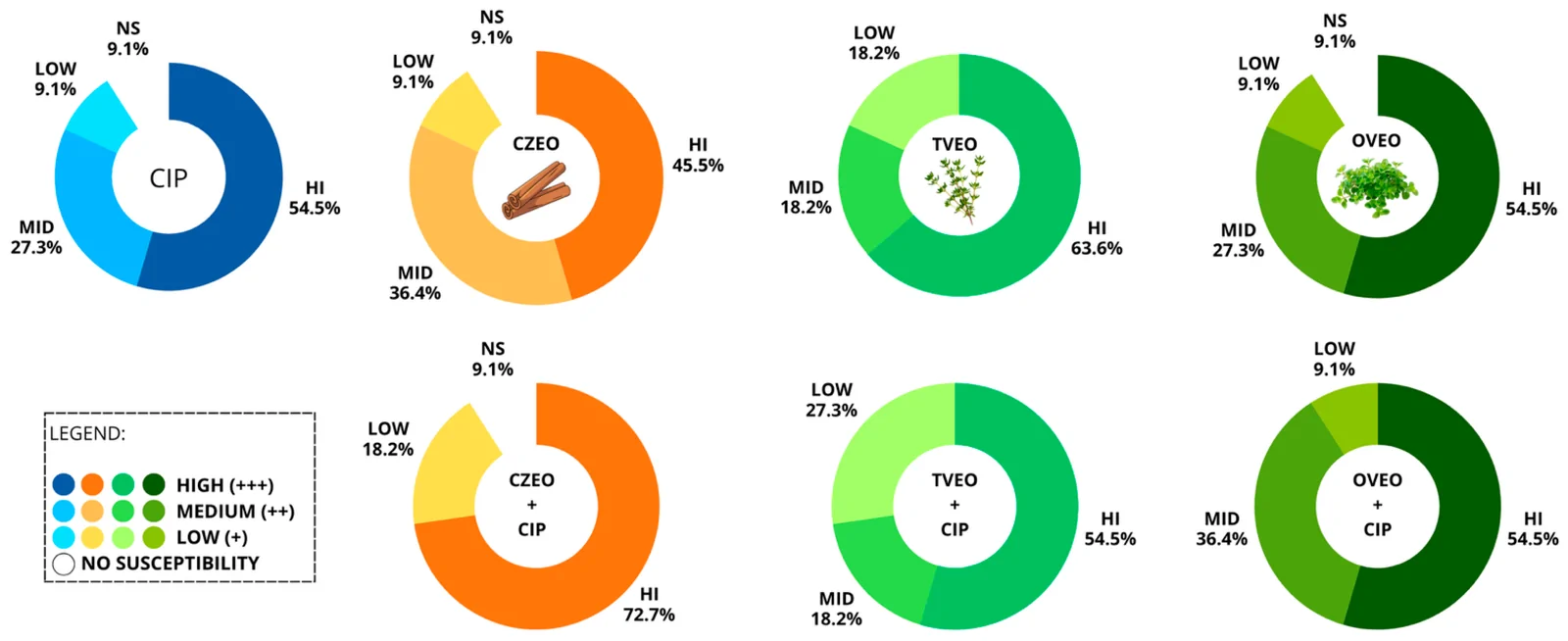
Configuration of the antibacterial activity profile on bacteria obtained from vaginal swabs.

*Vaginal swab.* Data on microorganism identification, antibiogram, aromatogram, and the combined effects of EOs and CIP for the 11 vaginal swab samples included in the study are summarized in Table 2. Figure 3 presents the results for *E. faecalis*, whereas Figure 2 provides a schematic overview of susceptibility patterns, as previously described for urine culture isolates. In contrast to the urine samples, a reversal in the prevalence of bacterial species was observed: *E. faecalis* was detected in 73% of samples, and *E. coli* in the remaining 27%.

The antibiogram analysis showed that 91% of the isolates were susceptible to **CIP**, with varying degrees of susceptibility: 9.1% were classified as low-susceptible, 27.3% as intermediately susceptible, and 54.5% as highly susceptible (yellow flows in Figure 2).

The aromatogram analysis showed that all isolates were susceptible to at least one EO, though to varying degrees. As shown in Figure 3, for CZEO, 45.5% of isolates were highly susceptible, 36.4% intermediately susceptible, and 9.1% low susceptible, whereas 9.1% were classified as not susceptible. For OVEO, 54.5% of isolates were highly susceptible, 27.3% intermediately susceptible, and 9.1% low susceptible, while 9.1% showed no activity. All isolates were susceptible to TVEO, with 63.6% classified as highly susceptible, 18.2% as intermediately susceptible, and 18.2% as low susceptible. The combined activity of **CIP** with **OVEO** or **TVEO** resulted in 100% susceptibility among the tested isolates, whereas the combination with **CZEO** showed a slightly lower overall efficacy (90%).

As observed in urine culture isolates, susceptibility to the combinations varied among isolates. For the OVEO-CIP combination, 54.5% of isolates were highly susceptible, 36.4% were intermediately susceptible, and 9.1% were low-susceptible. For the TVEO-CIP combination, 54.5% of isolates were highly susceptible, 18.2% were intermediately susceptible, and 27.3% were low-susceptible. Finally, for the CZEO-CIP combination, 72.7% of isolates were highly susceptible, and 18.2% were low-susceptible. A summary of all samples is shown in Figure 2, which plots both sample types on the same graph. Overall, only two isolates exhibited greater susceptibility to the EOs alone than to CIP alone (samples 8, 22, and 23, represented by the green flow in Figure 2); among these isolates, one was non-susceptible to CIP alone but susceptible to the tested EOs. More often, susceptibility with the EO–CIP combinations exceeded that with either CIP or the individual EOs alone; these cases, accounting for 27% of the samples, were classified as “Susceptibility to CIP enhanced by EOs” (cyan flows in Figure 2). In four cases (samples 18, 19, 29, and 30, represented by the grey flow), a “Susceptibility to CIP reduced by EOs” profile was observed, characterized by lower susceptibility to the combination than to the individual treatments (either one of the EOs or CIP alone). In these cases, which represent 13% of the total, microorganisms probably adopt alternative strategies to evade treatment.

Only one isolate showed higher susceptibility to the EOs alone than to CIP alone (green flow in Figure 2). More often, susceptibility with the EO-CIP combinations exceeded that with either CIP or the individual EO alone; these cases were classified as “ Susceptibility to CIP enhanced by EOs” (cyan flows in Figure 2). The term “Susceptibility to CIP highly enhanced by EOs” was used to describe the case (only one *E. coli* isolate) in which susceptibility to the combination markedly exceeded that observed for both CIP and EO alone. Conversely, the term “Susceptibility to CIP reduced by EOs” was assigned to cases in which susceptibility to the combination was lower than that observed for the individual treatments (EOs or CIP alone).

### 2.2. Ex Vivo Evaluation of Guinea Pig Bladder Contractility

To verify the EOs’ target activities on bladder spontaneous and induced contractility, functional studies of guinea pig bladder smooth muscle were performed.

**Figure 4:**
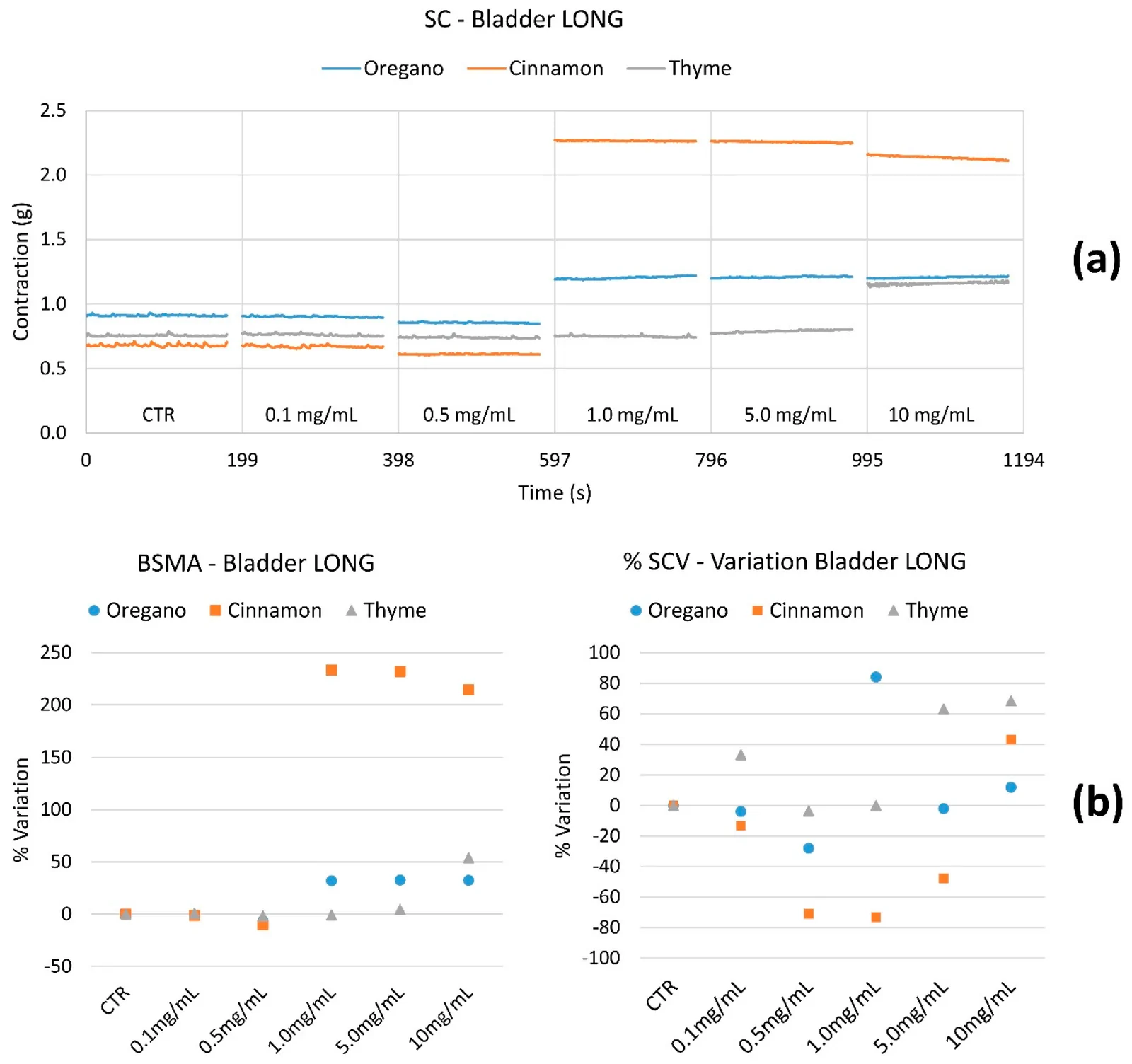
Experimental original recording of the concentration–response curve of **CZEO** (*Cinnamomum zeylanicum* Nees), **OVEO** (*Origanum vulgare* L.), and **TVEO** (*Thymus vulgaris* L.), on spontaneous bladder basal longitudinal contractility. (**a**) Spontaneous Contraction (SC) signals for each concentration; (**b**) Basal Spontaneous Motor Activity (BSMA) and Spontaneous Contraction Variability (SCV) percentage variations.

**Figure 5:**
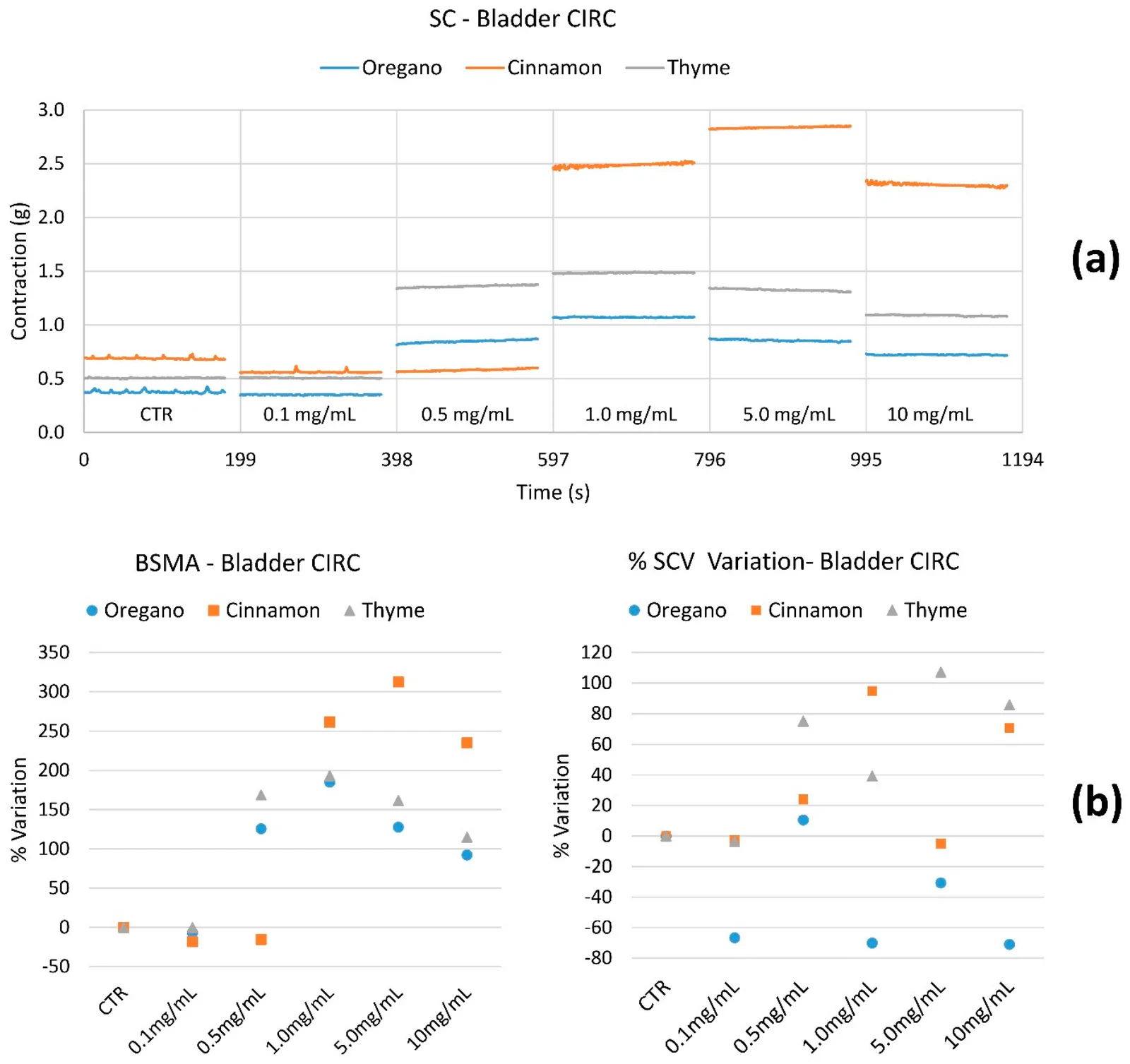
Experimental original recording of the concentration–response curve of **CZEO** (*Cinnamomum zeylanicum* Ness), **OVEO** (*Origanum vulgare* L.) and **TVEO** (*Thymus vulgaris* L.), on spontaneous bladder basal circular contractility. (**a**) Spontaneous Contraction (SC) signals for each concentration; (**b**) Basal Spontaneous Motor Activity (BSMA) and Spontaneous Contraction Variability (SCV) percentage variations.

*Spontaneous contractility.* The parameters of interest (BSMA, % SCV) were assessed by measuring muscle tone (related to tissue distension) under different conditions. The standard deviation (SCV), an index of variability, is therefore directly related to tissue oscillations. Figure 4 and Figure 5 show five minutes of original tracing in the different conditions (SC) and the percentage changes in tone (BSMA) and variability (% SCV), respectively, for the longitudinal and circular muscles. MCA values for both longitudinal and circular smooth muscle showed statistically significant differences (*p* < 0.05) at all concentrations tested.

At low concentration (0.1 mg/mL), **OVEO** and **CZEO** induced mild tissue relaxation, improving distensibility and functional contractile coordination, which may translate into improved voiding dynamics. In contrast, **TVEO** did not show significant effects at this concentration.

At 0.5 mg/mL, all three essential oils produced positive effects on the longitudinal muscle layer. However, **OVEO** and **TVEO** significantly increased tone in the circular muscle layer.

Spontaneous motility, closely associated with bladder discomfort and pain, was evaluated using percentage changes in the standard deviation of contractile oscillations (SCV). A reduction in oscillation amplitude was observed in the circular muscle layer across all essential oils at concentrations up to 0.5 mg/mL. Specifically, at 0.1 mg/mL, SCV decreased by −67% with **OVEO**, −3% with **CZEO**, and −4% with **TVEO**.

In the longitudinal layer, **OVEO** and **CZEO** maintained beneficial modulatory effects up to 0.5 mg/mL. In contrast, **TVEO** induced a modest increase in spontaneous variability at 0.1 mg/mL (+33%), followed by a decrease at 0.5 mg/mL.

Overall, at lower concentrations (0.1 mg/mL), **OVEO** and **CZEO** appear to promote tissue relaxation and enhance functional voiding capacity.

Collectively, these findings suggest that **OVEO** and **CZEO**, but not **TVEO**, exert favorable modulatory effects on spontaneous bladder motility at lower concentrations, thereby reducing contractile oscillations linked to bladder discomfort and pain.

*Induced contractility*. The three essential oils were evaluated for spasmolytic activity against potassium-induced contraction (80 mM), with nifedipine as a positive control. All data are summarized in Table 3. OVEO and TVEO exhibited intrinsic activity exceeding 50% and comparable potency, whereas CZEO showed intrinsic activity not exceeding 30%. This pattern clearly distinguishes CZEO from OVEO and TVEO in spasmolytic efficacy. Under the same experimental protocol, nifedipine reduced potassium-induced contraction by 79%, demonstrating nanomolar potency. Overall, these results indicate that OVEO and TVEO possess moderate spasmolytic activity against depolarization-induced bladder contraction. In contrast, CZEO exhibits a markedly weaker effect, suggesting distinct mechanisms or lower efficacy in modulating calcium-dependent smooth muscle contractility.

### 2.3. Antioxidant Activity

Given the role of oxidative stress and inflammation in the pathophysiology of urinary tract infections, the antioxidant capacity of the tested essential oils was evaluated as a complementary property that may contribute to their overall effect.

The DPPH radical-scavenging activity of the essential oils, expressed as IC_50_, is reported in Table 4, with ascorbic acid (AA) used as the reference compound. All essential oils exhibited concentration-dependent antioxidant activity. CZEO (0.02 mg/mL) showed the highest antioxidant activity (IC_50_ 0.02 mg/mL), whereas OVEO and TVEO were 15.5-fold less potent, with IC_50_ values of 0.31 mg/mL and 0.23 mg/mL, respectively.

### 2.4. Chemoinformatics and Bioinformatics Analysis

Information on drug mechanisms, molecular activities, protein functions, and biological pathways was retrieved from the widely used databases DrugBank [21], ChEMBL [22], UniProt [23], and KEGG [24].

The previously published list of 41 molecules [18], which reports the composition of the three EOs, was used to retrieve corresponding identifiers from ChEMBL (version 36). Only 33 molecules had a ChEMBL ID, and 29 of them had at least one type of biological activity reported. The retrieved data were filtered to focus on the target organisms, *E. coli* and *E. faecalis*. The majority of activity types were related to general antibacterial activity, without a specific target, including MIC or MIC50. Since this information is not directly correlated with the predicted protein–ligand complementarity, and the targets found were not present in the **CIP** pathways, the analysis was continued only with the predicted targets [18].

**Figure 6:**
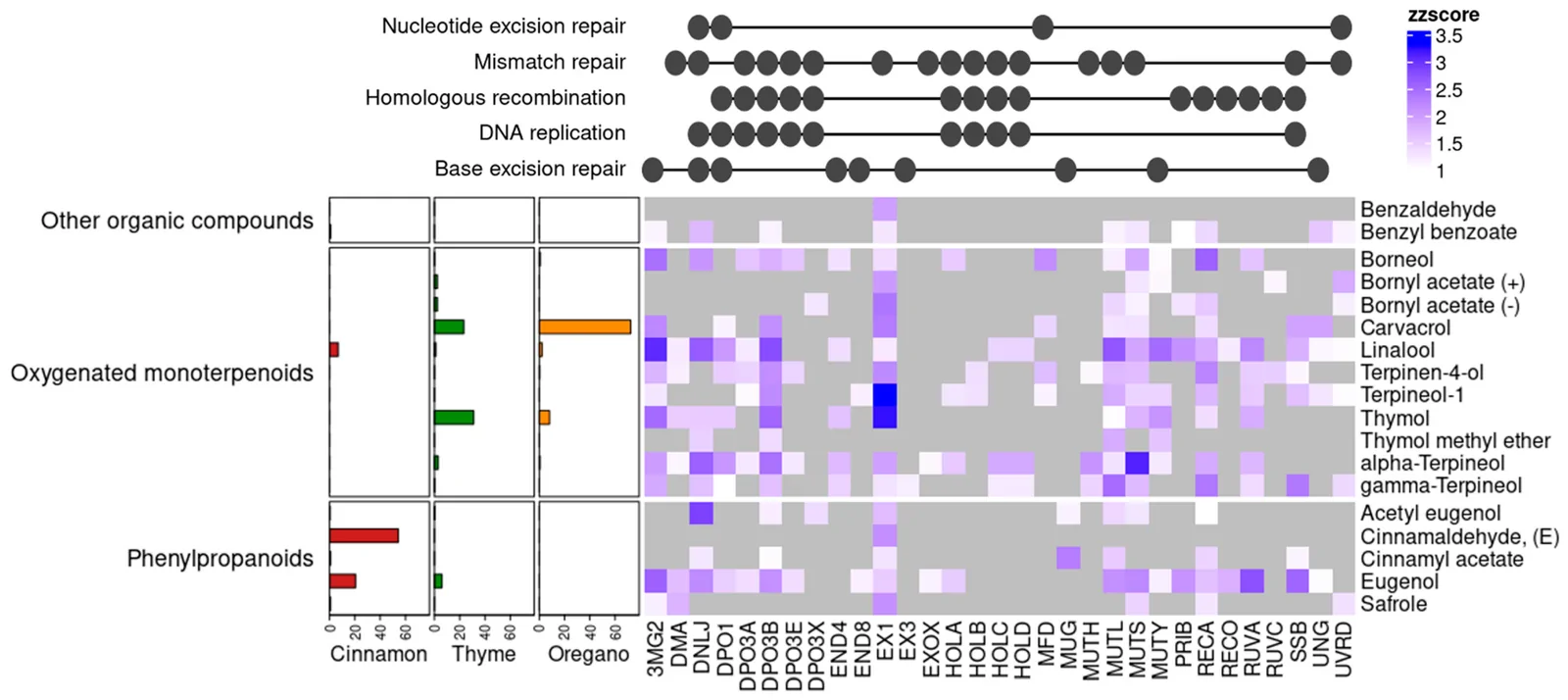
Target–ligand complementarity heatmap. The plot highlights genes involved in the same biological pathways as DNA gyrase; circles indicate the presence of genes in those pathways. The heatmap below associates the genes with the corresponding proteins, which are potentially druggable because they have a binding site for a small molecule. The heatmap reports the target–ligand pairs identified after normalization of the BioGPS scores. In the map, darker cells represent pairs with higher complementarity between the ligand and the corresponding binding pocket. Molecules are grouped by phytochemical classes, whereas compositional data are reported on the left as histograms. Molecules were not included in the heatmap whenever all their zzscore values were below 1.

According to DrugBank, the antibacterial activity of **CIP** is associated with inhibition of DNA gyrase subunit A, a bacterial protein involved in several biological pathways listed in Figure 6 (top). These pathways include additional genes whose corresponding protein structures are available in the Protein Data Bank archive and were therefore included in the BioGPS “pocketome” (as described in reference [18]). The pocketome is a large collection of protein cavities characterized using GRID Molecular Interaction Fields [25,26,27] and exploited by the BioGPS software [28,29] for several applications, including the evaluation of protein–ligand complementarity. Unfortunately, the available pocketome for *E. faecalis* was not sufficiently extensive to support a reliable analysis; therefore, subsequent investigations were limited to *E. coli*.

Previous studies [18,30] described a normalization procedure for analyzing BioGPS data in comparative multi-ligand/multi-target studies. In the present work, zzscore values were extracted for targets within the same biological pathways as DNA gyrase. Higher zzscore values indicate stronger predicted pocket–ligand complementarity, but a threshold of 1 was adopted as a symbolic cutoff to include weak target–ligand interactions in the study as well.

Based on these calculations, a shortlist of putative targets that interact with one or more EO constituents was obtained. Figure 6 presents the corresponding zzscore values as a heatmap, with darker cells indicating stronger predicted target–ligand interactions. This graphical tool helps identify the most frequent hits that engage the bacterial targets (the analysis identified several relevant targets, some of which are discussed further in the Discussion Section). From a molecular perspective, the heatmap reports interactions at the individual compound level, whereas the histograms on the left summarize each compound’s compositional contribution within the corresponding EO. Notably, compounds representing major EO constituents, including carvacrol, eugenol, linalool, and thymol, were predicted to interact with multiple targets. In contrast, (E)-cinnamaldehyde was predicted to interact exclusively with the protein target EX1.

## 3. Discussion

UTIs, particularly those affecting the lower urinary tract, are among the most common infectious diseases worldwide and can affect both women and men across age groups [31]. In most cases, infection begins with periurethral contamination by a uropathogen from the intestinal microbiota, followed by urethral colonization and ascent into the bladder. Uropathogens develop specialized virulence factors, including pili and flagella, and produce adhesins that facilitate migration and adherence to the urothelium, thereby enabling successful colonization of the lower urinary tract.

The pathogen’s ability to localize to the bladder epithelium, adapt to the urinary microenvironment, and evade host immune surveillance supports its survival and replication [32].

Prompt and appropriate treatment of UTIs is essential to confine the infection to the lower urinary tract and prevent progression to the upper urinary tract or systemic dissemination. In addition to pharmacological interventions, proper nutrition plays a key role in supporting urinary tract health by modulating the microbiota, enhancing immune defenses, and reducing susceptibility to infection.

*Escherichia coli* and *Enterococcus faecalis* are the most common pathogens causing cystitis [4,33]. In the bladder, *E. coli* uses type 1 fimbriae, with the FimH adhesin mediating binding to uroplakins and integrins, thereby facilitating stable attachment, invasion, and persistence [34]. The formation of intracellular bacterial communities (IBCs) enables *E. coli* to evade host defenses, resist antibiotics, and establish quiescent reservoirs that can reactivate in response to mechanical or traumatic stimulation [35]. *E. faecalis* relies on adhesion factors, including Ebp (Endocarditis and biofilm-associated pili), to colonize the urothelium and urinary catheters. Host-derived fibrinogen released during catheterization provides a substrate for adhesion and biofilm formation, contributing to bacterial persistence and inflammation in the urinary tract [36].

Fluoroquinolones, particularly ciprofloxacin, are widely used to treat cystitis by inhibiting DNA gyrase and topoisomerase IV, thereby disrupting bacterial replication and transcription [37,38]. However, repeated or inappropriate use drives resistance in *E. coli* through gyrA mutations and adaptive mechanisms, often resulting in multidrug-resistant strains [39,40,41,42]. These considerations underscore the need for complementary or alternative strategies to manage UTIs and limit antimicrobial resistance, including natural compounds that target pathogens no longer responsive to conventional therapies.

The identification of substances that can enhance inhibition with the combination of existing antibiotics represents a promising strategy to increase therapeutic efficacy, reduce effective dosages, and potentially limit the emergence of further resistance [43,44].

Analysis of clinical samples from urine cultures and vaginal swabs revealed a predominance of *E. coli* in urine isolates and of *E. faecalis* in vaginal swabs.

Antibiotic susceptibility testing revealed distinct patterns (Table 1 and Table 2), confirming the variability in antibiotic responsiveness observed in clinical isolates compared with previously studied ATCC (American Type Culture Collection) reference strains. These findings further underscore the dynamic and heterogeneous nature of antimicrobial susceptibility in real-world clinical settings.

EOs are widely recognized for their antibacterial properties; their biological activity is primarily attributed to bioactive constituents such as terpenes, terpenoids, and phenolic compounds, which can exert antimicrobial effects through multiple mechanisms [18].

**Figure 7:**
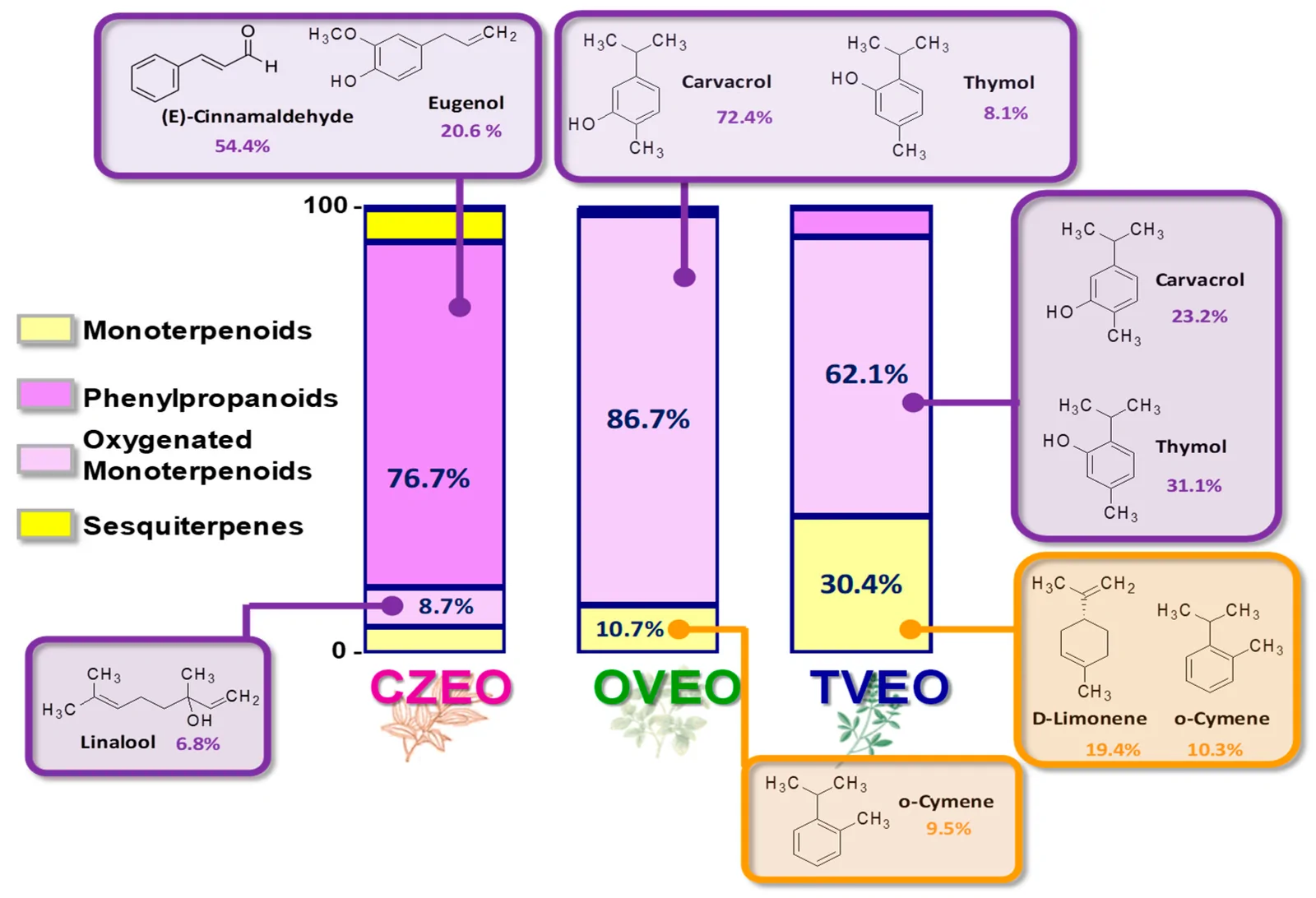
Major compounds (>5%) identified in **CZEO** (*Cinnamomum zeylanicum* Nees bark), **OVEO** (*Origanum vulgare* L. flowering tops), and **TVEO** (*Thymus vulgaris* L. flowering tops).

Chemical characterization of the previously investigated EOs revealed noteworthy similarities in their chemical profiles (Figure 7), especially among **OVEO** and **TVEO**, which overlap due to the high abundance of monoterpenoids, either oxygenated or not. In contrast, CZEO is largely composed of phenylpropanoids [18]. Supplementary Table S1 details the constituents of the tested Eos.

A combined chemo- and bioinformatics approach enabled the identification of putative molecular targets of the compounds present in the EOs; the structure-based pipeline detailed in our previous study [18] suggested that the three essential oils **CZEO**, **TVEO**, and **OVEO** may interfere with multiple bacterial metabolic processes and nucleic-acid-related pathways, thereby potentially enhancing antibiotic activity.

Notably, thyme and oregano EOs exhibited chemical overlap, which was confirmed in the predicted biological pathway profiles. Consistent with this convergence, the present study provided experimental support through comparable antibacterial effects observed in urine culture clinical isolates treated with these oils.

Here, we present a deeper investigation of previously published BioGPS data [18], focusing specifically on the biological pathways associated with DNA gyrase. This approach, which extends beyond the pathway significance emerging from gene set enrichment analysis (GSEA), enabled the identification of several putative intracellular targets that may be affected by EO constituents.

It is worth noting that the set of targets included in the analysis does not represent the entire bacterial genome. As previously reported [18], the BioGPS pocketome available for E. coli was estimated to cover approximately 42.7% of the bacterial proteome, leaving a large portion of the proteome unconsidered.

Among the most represented targets were proteins involved in DNA replication, mismatch repair, and homologous recombination. In particular, the MutS–MutL–MutH mismatch repair system showed predicted interactions with a relatively large number of EO constituents, especially MutS and MutL (14/18 compounds each), whereas MutH displayed a more limited interaction profile (3/18 compounds). The highest zzscore values were observed for linalool, α-terpineol, γ-terpineol, and eugenol. These proteins are essential for recognizing and repairing replication-associated mismatches and contribute to maintaining genome integrity [45,46].

Another highly represented target was Exonuclease I (ExoI, EX1), which interacted with 17 of the 18 EO constituents analyzed. The strongest predicted complementarity was observed for α-terpineol and thymol. ExoI is a single-stranded DNA-specific exonuclease involved in DNA repair and mutation avoidance pathways, and it is believed to participate in degrading the mismatched DNA strand after activation of the MutS–MutL–MutH complex [47,48]. The simultaneous targeting of MutS/MutL proteins and ExoI therefore suggests that EO constituents may interfere with multiple sequential steps of bacterial mismatch repair.

The BioGPS analysis also identified several DNA polymerase III (Pol III) subunits as potential EO targets, particularly DPO3B, which showed predicted interactions with 13/18 compounds, whereas DPO3A, DPO3E, and DPO3X interacted with 6/18, 4/18, and 2/18 compounds, respectively. Linalool, thymol, and α-terpineol displayed the highest zzscore values for these targets. DNA polymerase III is the major bacterial replicative polymerase and plays a central role in DNA replication and proofreading. In particular, the β subunit acts as the sliding clamp required for replication processivity, whereas the ε subunit provides proofreading through its 3′→5′ exonuclease function [49,50]. Predicted interactions involving these subunits therefore suggest possible interference with DNA synthesis and replication fidelity.

RecA was another highly represented target, interacting with 14/18 EO constituents. Borneol, γ-terpineol, and terpinen-4-ol showed the highest zzscore values. RecA is a key regulator of homologous recombination and the bacterial SOS response, promoting DNA repair and adaptation to replication stress and DNA damage [51,52]. Because RecA-mediated pathways are central to bacterial survival under genotoxic conditions, interfering with this protein may significantly impair bacterial stress responses.

Overall, these observations support the hypothesis that EO constituents may exert multitarget intracellular activity beyond membrane perturbation. The predicted interactions involve interconnected pathways associated with genome replication, mismatch repair, homologous recombination, and stress adaptation. In this context, simultaneous perturbation of DNA replication and DNA repair pathways may be particularly relevant to the mechanism of action of CIP, which targets DNA gyrase and induces replication stress and DNA damage. Therefore, impairment of downstream repair and recovery systems by EO constituents could contribute to the enhanced antibacterial activity observed in the disk-diffusion combination assay.

The results of experiments on clinical isolates are presented in Figure 1 and Figure 3, which highlight findings for *E. coli* and *E. faecalis*, the most prevalent microorganisms identified in urine cultures and vaginal swabs, respectively.

In urine-derived isolates, high susceptibility was observed to both ciprofloxacin and the three tested essential oils. Notably, combined treatment with ciprofloxacin and essential oils mitigated reduced responsiveness in less susceptible isolates, suggesting a potentially favorable combination effect.

Clinical evidence suggests that reduced susceptibility to CIP may be mitigated through combination therapy, either with other antibiotics or with bioactive natural compounds. Such combined approaches may enhance antibacterial efficacy and help overcome resistance mechanisms observed in clinical isolates [39]. This strategy aligns with the rationale for integrating conventional antibiotic therapy with bioactive natural compounds to enhance antibacterial efficacy, reduce the likelihood of resistance development, and potentially prevent progression of overt infection.

Accordingly, the present study was conceived as a preliminary preclinical investigation to characterize the antibacterial activity of these essential oils against clinical isolates and to explore their effects on bladder contractility. Specifically, we investigated the combined use of CIP and essential oils and observed several noteworthy findings. In most cases, in vaginal swab isolates, the combination treatment demonstrated greater efficacy than CIP alone.

Two CIP-resistant isolates were identified in this study, sample 1 from urine cultures (*E. coli*) and sample 23 from vaginal swabs (*E. faecalis*). Both isolates remained susceptible to the tested essential oils, and notably, this susceptibility was maintained under combination treatment, with resistance to ciprofloxacin not re-emerging. Although susceptibility varied, the combined approach consistently improved antibacterial responsiveness.

The recurrent nature of urinary tract infections and repeated antibiotic exposure not only promote the development of resistance but may also contribute to functional alterations in the bladder wall, including involvement of the urothelial epithelium [53].

It is well established that treatment of cystitis and associated lower urinary tract symptoms may also include spasmolytic agents, which do not treat the infection itself but help relieve symptoms such as urgency, frequency, pain, and bladder spasms. These include antimuscarinic drugs [54], β_3_-adrenoceptor agonists [55] and calcium modulators targeting voltage-dependent L-type calcium channels (Cav1.2), which play a crucial role in bladder contractility [56]. Calcium channels are central regulators of detrusor muscle contraction, acting both directly through voltage-gated calcium influx and indirectly via signaling pathways downstream of G-protein-coupled receptor activation. Therefore, modulation of intracellular calcium dynamics represents a key therapeutic target in the control of bladder wall hyperactivity and dysfunction [55,57]. In addition, certain calcium channel modulators may enhance the activity of antibacterial agents. For example, verapamil has been shown to inhibit P-glycoprotein (P-gp, also known as P-170), a membrane efflux transporter associated with multidrug resistance. Inhibition of this transporter may enhance intracellular drug accumulation and contribute to restoring sensitivity to conventional antibacterial agents [58]. Pain management in cystitis also commonly includes nonsteroidal anti-inflammatory drugs (NSAIDs), which help control inflammation-related symptoms and improve patient comfort [59,60,61]. However, prolonged use of NSAIDs is associated with relevant off-target effects, particularly involving the cardiovascular, gastrointestinal, and renal systems [59].

Pain associated with cystitis may also be linked to ion-channel dysregulation, including L-type voltage-gated calcium channels (Cav1.2), which are key regulators of nociceptive signaling [62]. These channels are central to the modulation of pain transmission and smooth muscle contractility. Accordingly, L-type calcium channel modulators have been explored as supportive therapeutic options for managing bladder dysfunction and associated symptoms [63]. Targeting this ancillary yet clinically relevant pathway may therefore provide additional therapeutic benefit alongside conventional antibacterial therapy. In this context, **OVEO** and **TVEO** were found to interfere with calcium influx in bladder cells, displaying an activity profile qualitatively comparable to that of nifedipine, albeit with expected differences in potency (Table 3).

These EOs, which contain thymol and carvacrol along with comparable amounts of o-cymene, exhibited spasmolytic activity, consistent with previous reports on extracts rich in o-cymene and related monoterpenes [64], suggesting a potential role in modulating detrusor contractility and alleviating bladder hyperactivity during urinary tract infections.

In the context of bladder functional regulation, the present results provide insight into the differential effects of EOs on detrusor activity. A concentration- and tissue layer-dependent modulation of bladder contractility was observed, with **OVEO** and **CZEO** showing a more favorable pharmacodynamic profile at lower doses by enhancing relaxation and reducing spontaneous activity. These effects may improve voiding efficiency and alleviate bladder discomfort, potentially supporting the management of UTIs by facilitating bladder emptying and reducing irritative symptoms. In contrast, TVEO appears comparatively less effective and, in some conditions, may exert less beneficial modulatory actions.

In addition to the multi-target mechanisms described above, several bioactive constituents present in these essential oils have well-documented pharmacological properties that may further support their therapeutic potential. Eugenol exhibits anti-inflammatory and analgesic effects [65]; (E)-cinnamaldehyde is recognized for its anti-inflammatory and antioxidant activity [66,67]; linalool displays anti-inflammatory, analgesic, and spasmolytic properties [68,69,70]; o-cymene and D-limonene are associated with anti-inflammatory and antioxidant effects [71]; carvacrol exerts anti-inflammatory activity [72]; and thymol is widely known for its antiseptic properties [73]. Collectively, these complementary bioactivities may potentiate the observed antimicrobial and symptom-modulating effects.

However, despite their documented therapeutic properties, essential oils are not without adverse effects, particularly when used for self-medication or in vulnerable populations [74]. Further studies, including in vivo efficacy, pharmacokinetic, safety/toxicological, and ultimately clinical investigations, are required before any conclusions regarding their therapeutic potential can be drawn. For this reason, developing controlled and standardized formulations is paramount. In particular, solid dosage forms may offer advantages in stability, bioavailability, and patient compliance, and may reduce the overall drug burden, provided that their use remains under appropriate medical supervision [74].

In addition to modulating molecular targets associated with conventional cystitis therapy, the EOs also demonstrated significant antioxidant activity, with CZEO showing the highest activity, followed by TVEO and **OVEO**. This property may further support their potential use in the management of UTIs, as attenuating oxidative stress could help limit urothelial damage and inflammation associated with infection.

The integration of antimicrobial, spasmolytic, and antioxidant properties within a single multitarget framework represents a promising step toward more comprehensive and sustainable approaches to cystitis management. In this context, the present findings provide preliminary evidence supporting the potential of these essential oils as a source of bioactive compounds and as a basis for developing standardized essential-oil-derived preparations, warranting further investigation to elucidate their mechanisms of action, efficacy, and safety.

## 4. Materials and Methods

### 4.1. Essential Oils (EO) Chemical Composition

The essential oils of *Cinnamomum zeylanicum* Ness bark (**CZEO**), *Origanum vulgare* L. flowering tops (**OVEO**), and *Thymus vulgaris* L. flowering tops (**TVEO**) used in this study were those contained in the solid formulations marketed as Aromatoil^®^ (manufactured by Coima, Bastia (RA), Italy) and supplied by BIO-LOGICA, Via della Zecca 1, 40100, Bologna, Italy. The solid formulations were obtained as previously described [75], and their chemical composition was recently published [18].

### 4.2. Antimicrobial Activity

#### 4.2.1. Isolation and Identification of Bacterial Strains

The strains obtained from human urine cultures (19 samples) and vaginal swabs (11 samples) were isolated and identified in the Microbiological Section of Laboratorio Valsambro, Bologna, Italy, using conventional physiological and morphological methods [76,77]. The samples used for the study were residual diagnostic materials collected and separated before disposal after clinical testing and not required for subsequent investigations. The samples were randomly assigned a unique code using a computerized tool that ensured complete anonymity. The strains were identified in single cultures as *Enterococcus faecalis* or *Escherichia coli* [78].

The isolated colonies were cultured and maintained on Mueller–Hinton agar at 35 ± 2 °C throughout the experimental procedures, which included treatment with each EO and with ciprofloxacin (CIP), used as a positive control, either alone or in combination.

Clinical specimens were processed using Liofilchem^®^ Chromatic™ Detection (Liofilchem S.r.l., Roseto degli Abruzzi, Teramo, Italy), a chromogenic medium designed for the enumeration and presumptive identification of microorganisms directly from clinical and non-clinical samples. For urine cultures, a disposable calibrated 1 μL inoculating loop was used to transfer the specimen onto the agar plate. Vaginal swabs were directly streaked onto the agar surface using the same isolation technique. In both cases, the inoculation procedure was performed to obtain well-isolated colonies. Plates were incubated aerobically at 35 ± 2 °C for 18–24 h. Presumptive identification was based on the colony characteristics observed on the chromogenic medium.

Molecular identification was additionally performed by PCR to confirm the identity of the isolates.

*Escherichia coli* ATCC 700728 and *Enterococcus faecalis* ATCC 29212 were included as reference control organisms.

#### 4.2.2. Antibacterial Ciprofloxacin Susceptibility

The antibacterial **CIP** sensibility was assessed using the Kirby-Bauer disk diffusion method in accordance with guidelines from the Clinical and Laboratory Standards Institute (CLSI) [79]. Microorganisms were classified as susceptible or resistant to the positive control **CIP**: not susceptible (/), low susceptible (+: zone of inhibition < 4 mm), intermediate susceptible (++: zone of inhibition between 4 and 10 mm), or high susceptible (+++: zone of inhibition > 10 mm), respectively, as previously described [80].

The Minimal Inhibitory Concentration (MIC) values were determined for six replicates using the microdilution method [81]. We previously demonstrated [75] that the excipient mixture alone (pregelatinized corn starch, soy lecithin, ascorbic acid, calcium carbonate, levilite, vegetable magnesium stearate) did not inhibit bacterial colonies or the principal bacterial species constituting the urogenital microbiota. In contrast, **CIP** inhibited the growth of bifidobacteria and lactobacilli.

Vaginal microbiota samples were collected using a genital swab or brush to test for the presence of pathogens. Data were analyzed using IBM SPSS (Statistical Package for the Social Sciences) software (version 19, IBM SPSS Inc., Chicago, IL, USA). All samples and control groups were compared at the 95% confidence interval, as previously described [82].

#### 4.2.3. Aromatogram of Essential Oils

The susceptibility of antibacterial essential oils (**CZEO**, **OVEO**, and **TVEO**) was assessed using a conventional disc diffusion method (aromatogram) previously described [83].

Sterile paper disks (6 mm diameter, TermoFisher, Milano, Italy) were impregnated with 2 μL of essential oil (corresponding to 220 mg/mL for **CZEO**, 240 mg/mL for **OVEO** and 190 mg/mL for **TVEO**, respectively) and placed on Mueller–Hinton agar plates (TermoFisher, Milano, Italy), previously inoculated with a 10^8^ CFU/mL (Colony Forming Unit per milliliter) suspension of each identified clinical isolate. After 24 h of incubation at 35 ± 2 °C, the diameter of the inhibition zones was measured, and the degree of susceptibility was recorded according to the symbols described above. The procedure was repeated for each essential oil.

#### 4.2.4. Combined Effect of Ciprofloxacin and Essential Oils

On plates containing the isolated and identified bacterial strains, a standard CIP disk (5 μg) was placed. A 2 μL drop of the corresponding essential oil (0.44 mg for **CZEO**, 0.48 mg for **OVEO**, and 0.38 mg for **TVEO**, respectively) was applied directly onto the antibiotic disk. Plates were incubated aerobically at 35 ± 2 °C for 24 h. Inhibition zones were then measured, and susceptibility was recorded as previously described [83]. This procedure was repeated for all three essential oils.

### 4.3. Ex Vivo Bladder Contractility Assessment

#### 4.3.1. Animals

Guinea pigs of either sex (200–400 g) obtained from Charles River (Calco, Como, Italy) were used. Animal care and experimental procedures were conducted in accordance with European and Italian legislation governing the use and care of laboratory animals (EU Directive 2010/63/EU and Italian Legislative Decree 26/2014) and in compliance with the ARRIVE 2.0 guidelines [84]. All experimental protocols were approved by the University of Bologna Animal Welfare Body and authorized by the Italian Ministry of Health (Authorization No. 2DBFE.N.YEV, December 2023). Animals were housed for a 7-day acclimatization period under controlled conditions (12 h light/12 h dark cycle, 22 °C, 60% relative humidity), with food and water available ad libitum.

After the explant, the urinary bladder was rapidly mounted under a suitable resting tension in a 15 mL organ bath containing an appropriate physiological salt solution (PSS) that was consistently warmed (see below) and buffered to pH 7.4 by saturation with 95% O_2_–5% CO_2_ gas.

*Guinea-pig urinary bladder.* The method was described by Micucci et al. [85]. Briefly, the urinary bladder was cut into two strips and mounted in an organ bath at 34 °C under a tension of 1 g, containing Krebs solution of the following composition (mM): NaCl 95, KCl 4.7, CaCl_2_ 2.50, MgSO_4_ 1.0, KH_2_PO_4_ 1.2, NaHCO_3_ 25, and glucose 10.6, equilibrated with 95% O_2_–5% CO_2_ gas at pH 7.4. The tissues were allowed to stabilize for 90 min. Tension was recorded isometrically.

#### 4.3.2. Spontaneous Contractility of the Urinary Bladder

Spontaneous contractility was studied using the previously described method [86,87]. Briefly, for the urinary bladder, traces of spontaneous contractions were continuously recorded using LabChart Software (ADInstruments, Bella Vista, New South Wales, Australia). After the equilibration period (about 30 to 45 min, depending on the tissue), cumulative–concentration curves for EOs and the reference compound **Nifedipine** were constructed. At the end of each dose, the following parameters of the Spontaneous Contraction (SC) recording were evaluated, considering a 5-min stationary period: the Mean Contraction Amplitude (MCA), evaluated as the mean force value (g); the standard deviation of the force values over the period, as an index of the Spontaneous Contraction Variability (SCV); and the Basal Spontaneous Motor Activity (BSMA), as the percentage (%) variation in each mean force value (g) with respect to the control period. The spontaneous contraction variability (SCV) was referenced to the basal value to evaluate its percentage variation [87].

#### 4.3.3. Induced Contractility via L-Type Calcium Channel (LTCC)

Bladder spasmolytic activity mediated by calcium channels was studied using tissues contracted by 80 mM K^+^-concentration. Tension changes were recorded isometrically using a previously described method [87].

#### 4.3.4. Statistical Analysis

Data analysis of spontaneous contractility was performed in a post-processing phase using LabChart7 PRO Software (AD Instruments; Bella Vista, New South Wales, Australia). Comparisons of mean spontaneous contraction amplitudes (MCA) across concentrations were performed using one-way ANOVA with Bonferroni’s correction. Differences with *p* < 0.05 were considered statistically significant. To avoid errors caused by artifacts, the analysis period was selected by a skilled operator.

Data for induced contractility are reported as mean ± S.E.M. from *n* independent experiments and were analyzed using one-way ANOVA followed by Dunnett post hoc test (GraphPad Prism version 9.0, GraphPad Software, San Diego, CA, USA). Differences with *p* < 0.05 were considered significant. The potency defined by IC_50_ was calculated from concentration–response curves (essential oils) or log-concentration–response curves (**Nifedipine**) (Probit analysis using Litchfield and Wilcoxon [20] or GraphPad Prism^®^ [88,89]).

### 4.4. Antioxidant Activity via DPPH Radical Scavenging Assay

As previously described [90], radical-scavenging activity was evaluated using the 2,2-diphenyl-1-picrylhydrazyl (DPPH) assay. DPPH was prepared at 100 µM in methanol. Essential oils were solubilized in an aqueous Tween solution and then diluted in DMSO to achieve concentrations suitable for IC_50_ determination. For each test, 0.1 mL of sample was added to 2.9 mL of DPPH solution, incubated for 30 min at 25 °C in the dark, and absorbance was measured at 517 nm. A sample blank (2.9 mL methanol + 0.1 mL sample, without DPPH) was included to correct for sample turbidity and intrinsic absorbance. Radical-scavenging potency was calculated from IC_50_ values obtained by nonlinear regression of inhibition (%) versus concentration. Ascorbic acid served as a positive control [91].

### 4.5. Chemoinformatics and Bioinformatics

The chemical compositions of EOs were obtained from a previous study [16], along with the corresponding PubChem compound identifiers (CIDs). These CIDs were mapped to ChEMBL molecular identifiers using UniChem data [92] and used to query the ChEMBL database (release 36) [22] to retrieve activity data against *E. coli* and *E. faecalis*. For each compound, its putative bacterial targets were obtained from [18], along with their predicted activity score (zzscore).

To investigate the combined mechanism of action of **CIP** and EOs, the following steps were performed. First, the **CIP** targets in *Escherichia coli* (strain K12) were retrieved from the DrugBank database [21]; next, the identifiers of the biological pathways associated with these targets were retrieved from UniProt [23] (P0AES4) and KEGG (eco:b2231) [24].

## 5. Conclusions

The present findings demonstrate that the three EOs exhibit antibacterial activity against clinical isolates, albeit with distinct susceptibility profiles. This variability further supports the concept that pathogens within the host environment undergo adaptive changes that may reduce responsiveness to conventional antibiotics, particularly after prolonged exposure.

Notably, the EOs retained antibacterial activity even when isolated, showing reduced susceptibility to **CIP**. When administered alone, the EOs displayed measurable antibacterial effects, and in several cases, their combination with **CIP** enhanced overall antimicrobial efficacy, suggesting potentially favorable combination effect.

Computational analysis further indicated that EOs constituents may target multiple intracellular pathways involved in DNA replication, mismatch repair, homologous recombination, and bacterial stress responses. Such a multitarget mechanism may offer an important advantage for combinatorial therapeutic strategies, either through the co-administration of different EOs or by using them as adjuvants to conventional antibiotics. In this context, EOs-based approaches may help improve therapeutic efficacy and could serve as a complementary strategy to counteract the growing global challenge of antibiotic resistance [93].

Given their natural origin and established use in the food and nutraceutical fields, these EOs may also warrant further investigation as functional ingredients or dietary adjuvants that could support urinary tract health and help prevent or manage urinary tract infections, as complementary therapies in human health, offering a safe and natural alternative to traditional treatments. However, despite challenges related to the efficacy and safety of essential oils, the results demonstrate the potential of these compound blends in patients [94]. To fully investigate their therapeutic potential, further clinical research and carefully planned studies are needed, especially to develop appropriate formulations that increase bioavailability and minimize side effects [95].

**Figure 8:**
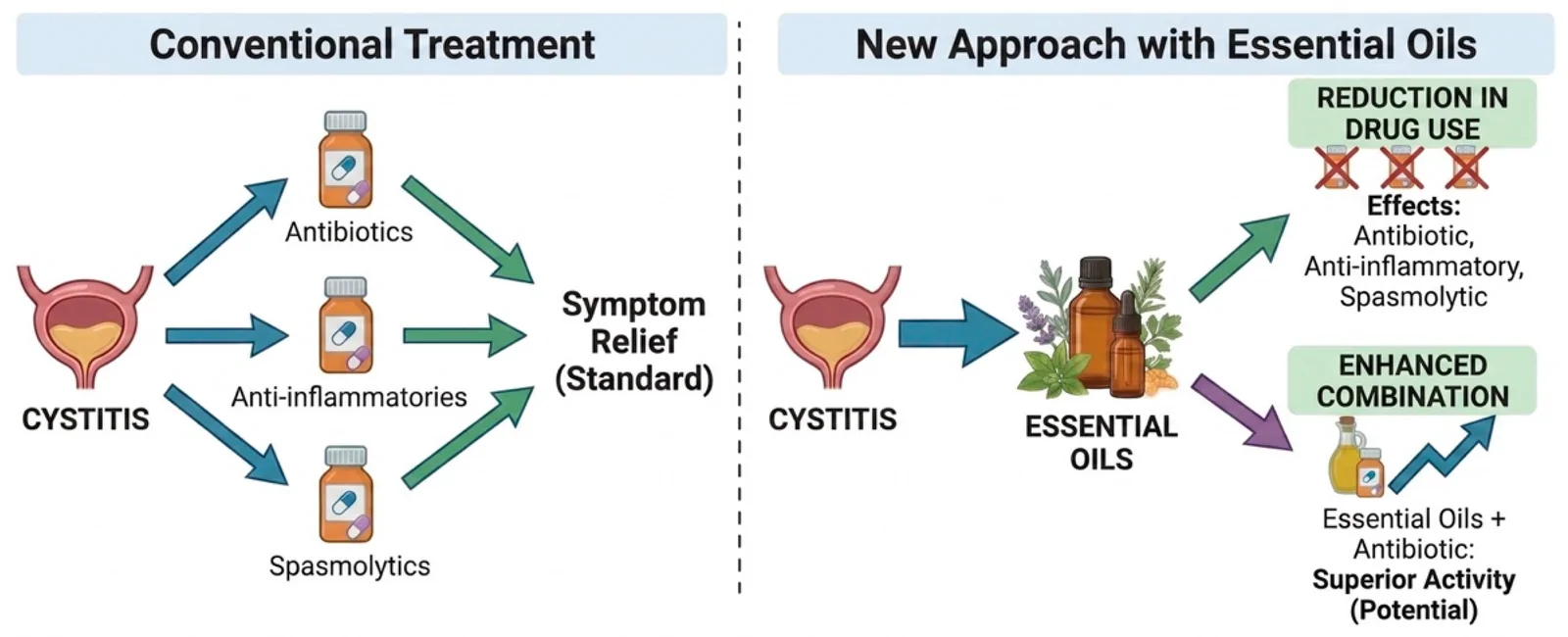
Outlook of the network target of the studied EOs.

In addition to their antibacterial activity, the investigated EOs may modulate key nodes within cystitis-related therapeutic networks that are also targeted by conventional drugs. In particular, in the ex vivo guinea pig bladder model, **OVEO** and **CZEO** produced mild, favorable modulatory effects on spontaneous bladder motility at lower concentrations, whereas **OVEO** and **TVEO** showed moderate spasmolytic activity against potassium-induced contraction, supporting a differential modulatory effect of the three essential oils on bladder smooth muscle function. Their spasmolytic effects, mediated through modulation of L-type calcium channels, may interfere with both spontaneous and induced bladder contractility. Through this mechanism, EOs could potentially contribute to the control of lower urinary tract symptoms and reduce the need for adjunctive spasmolytic therapies (Figure 8), in line with the “multicomponent multitarget” paradigm characteristic of these mixtures of chemical compounds.

## Figures and Tables

**Table 1:** Antibacterial activity of essential oils against clinical isolates from human urine cultures, evaluated by the Kirby–Bauer disk diffusion method.

Sample	Bacterial Isolate	CIP Susceptibility ^1^	MIC ^2^ (μg/mL)	EO ^3^ Susceptibility		Combination Susceptibility ^4^
**1**	*E. coli*	/	1.00	CZEO	++	++
				OVEO	++	+++
				TVEO	+++	+++
**2**	*E. coli*	++	0.023	CZEO	+++	+++
				OVEO	++	+++
				TVEO	+	+++
**3**	*E. coli*	++	0.012	CZEO	++	+++
				OVEO	+++	+++
				TVEO	+++	+++
**4**	*E. coli*	++	2.00	CZEO	+	+++
				OVEO	++	++
				TVEO	+	+++
**5**	*E. coli*	++	0.016	CZEO	+	+++
				OVEO	+++	+++
				TVEO	+++	+++
**6**	*E. coli*	++	0.023	CZEO	+	+++
				OVEO	++	+++
**7**	*E. coli*	++	0.012	CZEO	++	+++
				OVEO	++	+++
				TVEO	++	+++
**8**	*E. coli*	++	0.75	CZEO	++	++
				OVEO	+++	+++
				TVEO	++	++
**9**	*E. coli*	+++	0.19	CZEO	++	+++
				OVEO	+++	+++
				TVEO	+++	+++
**10**	*E. coli*	+++	1.00	CZEO	+++	+++
				OVEO	++	+++
				TVEO	+++	+++
**11**	*E. coli*	+++	1.50	CZEO	+++	+++
				OVEO	+++	+++
				TVEO	+++	+++
**12**	*E. coli*	+++	0.016	CZEO	++	+++
				OVEO	+++	+++
				TVEO	+	+++
**13**	*E. coli*	+++	0.016	CZEO	++	+++
				OVEO	+++	+++
				TVEO	+++	+++
**14**	*E. coli*	+++	0.75	CZEO	++	+++
				OVEO	+++	+++
				TVEO	+++	+++
				TVEO	++	+++
**15**	*E. coli*	+++	0.016	CZEO	++	+++
				OVEO	++	+++
				TVEO	+++	+++
**16**	*E. coli*	+++	1.00	CZEO	+	+++
				OVEO	+++	+++
				TVEO	++	+++
**17**	*E. feacalis*	+++	0.023	CZEO	+++	+++
				OVEO	+++	+++
				TVEO	+++	+++
**18**	*E. coli*	+++	0.25	CZEO	++	+++
				OVEO	+++	++
				TVEO	+++	+++
**19**	*E. coli*	+++	0.25	CZEO	++	+++
				OVEO	+++	++
				TVEO	++	+++

^1^ **CIP** (ciprofloxacin) 5 μg of ciprofloxacin. ^2^ MIC: Minimum Inhibitory Concentration. ^3^ EO: Essential Oil. **CZEO** (*Cinnamomum zeylanicum* Ness), **OVEO** (*Origanum vulgare* L.) and **TVEO** (*Thymus vulgaris* L). ^4^ Combination assay performed using 2 μL of essential oil added to a 5 μg ciprofloxacin disk. /: no antibacterial activity; +, zone of inhibition less than 4 mm; ++, zone of inhibition between 4 and 10 mm; +++, zone of inhibition greater than 10 mm.

**Table 2:** Antibacterial activity of ciprofloxacin and essential oils, alone or in combination, against clinical isolates obtained from vaginal swabs, evaluated by the Kirby–Bauer disk diffusion method.

Sample	Bacterial Isolate	CIP Sensibility ^1^	MIC ^2^ (μg/mL)	EO ^3^ Sensibility		Combination Sensibility ^4^
**20**	*E. coli*	++	1.00	CZEO	+++	+++
				OVEO	/	+++
				TVEO	+++	+++
**21**	*E. faecalis*	++	0.064	CZEO	+++	+++
				OVEO	+++	+++
				TVEO	+++	+++
**22**	*E. faecalis*	+	8.00	CZEO	++	+
				OVEO	++	+
				TVEO	+++	+
**23**	*E. faecalis*	/	4.00	CZEO	+++	+
				OVEO	+++	++
				TVEO	+	++
**24**	*E. coli*	+++	0.012	CZEO	+++	+++
				OVEO	+++	+++
				TVEO	+++	+++
**25**	*E. coli*	+++	0.25	CZEO	++	+++
				OVEO	+++	+++
				TVEO	+++	+++
**26**	*E. faecalis*	++	0.38	CZEO	/	/
				OVEO	++	++
				TVEO	++	++
**27**	*E. feacalis*	+++	0.12	CZEO	+	+++
				OVEO	++	+++
				TVEO	++	+++
**28**	*E. feacalis*	+++	0.016	CZEO	++	+++
				OVEO	+	+++
				TVEO	+	+++
**29**	*E. faecalis*	+++	1.00	CZEO	+++	+++
				OVEO	+++	++
				TVEO	+++	+
**30**	*E. faecalis*	+++	0.50	CZEO	++	+++
				OVEO	+++	++
				TVEO	+++	+

^1^ **CIP** (ciprofloxacin) 5 μg of ciprofloxacin. ^2^ MIC: Minimum Inhibitory Concentration. ^3^ EO: Essential Oil. **CZEO** (*Cinnamomum zeylanicum* Nees), **OVEO** (*Origanum vulgare* L.), and **TVEO** (*Thymus vulgaris* L.). ^4^ Combination assay performed using 2 μL of essential oil added to a 5 μg ciprofloxacin disk. /: no antibacterial activity; +, zone of inhibition less than 4 mm; ++, zone of inhibition between 4 and 10 mm; +++, zone of inhibition greater than 10 mm.

**Table 3:** Spasmolytic activity of Solid Essential oils in Guinea pig bladder preparations.

Sample	IA ^1^	EC_50_^2^	95% conf lim
***Cinnamomum zeylanicum***	23 ± 1.4 (*0.51* mg/mL)		
***Origanum vulgaris***	68 ± 0.7 (*0.51* mg/mL)	0.18	0.12–0.25
***Thymus vulgaris***	58 ± 0.9 (*0.51* mg/mL)	0.14	0.09–0.36
**Nifedipine**	79 ± 2.5 (50 μM) (*0.017* mg/mL)	0.0065 (μM) (*0.0022* μg/mL)	0.0042–0.0084 (μM) (*0.0014*–*0.0029* μg/mL)

^1^ **IA** (Intrinsic Activity) expressed as percent decrease in developed tension on isolated guinea-pig bladder, expressed as percent changes from the control (*n* = 5–6). The concentration indicated in brackets gave the maximum effect. ^2^ Calculated from concentration–response curves (EOs) or log concentration–response curves (**Nifedipine**) (Probit analysis by Litchfield and Wilcoxon [20] with *n* = 6–7). When the maximum effect was <50%, the EC_50_ values were not calculated.

**Table 4:** Antioxidant activity of essential oils obtained by the DPPH Assay.

Sample	IC_50_ ^1^ (mg/mL)
***Cinnamomum zeylanicum***	0.020 ± 0.010
***Origanum vulgaris***	0.310 ± 0.020
***Thymus vulgaris***	0.230 ± 0.090
**Ascorbic acid ^2^**	0.003 ± 0.0010

^1^ IC_50_, radical scavenging activity [concentration necessary for 50% reduction of 2,2-Diphenyl-1-Picrylhydrazyl (DPPH) radical]. Values were expressed as means ± SD. ^2^ Ascorbic Acid positive control.

## Data Availability

Data are available upon request to the corresponding author.

## Supplementary Materials

The following supporting information can be downloaded at https://www.mdpi.com/article/10.3390/antibiotics15090909/s1, Table S1: Chemical constituents of tested EOs.

## Abbreviations

The following abbreviations are used in this manuscript:

UTIs	Urinary Tract Infections
EOs	Essential Oils
CZEO	*Cinnamomum zeylanicum* Essential Oil
OVEO	*Origanum vulgare* Essential Oil
TVEO	*Thymus vulgaris* Essential Oil
CIP	Ciprofloxacin
NCCLS	National Committee for Clinical Laboratory Standards
MIC	Minimal Inhibitory Concentration
SPSS	Statistical Package for the Social Sciences
CFU	Colony Forming Unit
EEC	European Economic Community
WMA	World Medical Association
PSS	Physiological Salt Solution
SC	Spontaneous Contraction
MCA	Mean Contraction Amplitude
SCV	Spontaneous Contraction Variability
BSMA	Basal Spontaneous Motor Activity
SEM	Standard Error Mean
DPPH	2,2-diphenyl-1-picrylhydrazyl
DMSO	Dimethyl sulfoxide
IC_50_	50% Inhibitory Concentration
IA	Intrinsic Activity
IBCs	Intracellular Bacterial Communities
Ebp	Endocarditis and biofilm-associated pili
ATCC	American Type Culture Collection
NSAIDs	Non-Steroidal Anti-Inflammatory Drugs
