# Supplementary material for: Cinnamomum zeylanicum, Origanum vulgare, and Thymus vulgaris Essential Oils in Cystitis: An Alternative/Complementary Perspective
Source: Antibiotics (Basel). 2026 Sep 15;15(9):909. doi: 10.3390/antibiotics15090909 (PMC13603988; doi:10.3390/antibiotics15090909)
Supplement: Supplementary file 1 [file antibiotics-15-00909-s001.zip › antibiotics-4465168-supplementary.pdf]

Article

Cinnamomum zeylanicum, Origanum vulgare, and Thymus vulgaris Essential Oils in Cystitis: an alternative/complementary perspective

Supplementary table

Table S1. Chemical constituents of the essential oils of interest, from *Cinnamomum zeylanicum* Ness bark (**CZEO**), *Origanum vulgare* L. flowering tops (**OVEO**), and *Thymus vulgaris* L. flowering tops (**TVEO**). Values are expressed as percentage relative abundance. Compounds in bold represent the predominant constituents.[a]

**Compound**	*Cinnamomum zeylanicum* Ness **(%)**	*Origanum vulgare* L. **(%)**	*Thymus vulgaris* L. **(%)**
**Monoterpenes**			
**α-Pinene**	1.73	< 0.5	< 0.5
**o-Cymene**	1.41	**9.54**	**10.26**
**D-Limonene**	1.67	< 0.5	**19.35**
**Eucalyptol**	1.87	1.38	0.53
Linalool	**6.80**	2.17	1.13
Borneol	-	0.75	0.20
**α-Terpineol**	--	0.58	2.94
**Bornyl acetate**	--	--	2.33
**Terpineol-1**	-	-	0.10
**Terpinen-4-ol**	-	0.39	0.10
**Thymol**	--	**8.08**	**31.08**
**Carvacrol**	--	**72.36**	**23.23**
**Thymol methyl ether**	-	0.18	-
**y-Terpineol**	-	-	0.29
**Phenylpropanoids**			
**Acetyl eugenol**	0.33	-	0.39
**Cinnamaldehyde (E)**	**54.40**	--	--
**Cinnamyl acetate**	0.77	-	-
**Eugenol**	**20.55**	--	5.97
**Safrole**	0.65	-	-
**Sesquiterpenes**			

**Caryophyllene**	**6.73**	0.52	< 0.5
**Other organic compounds**			
**Benzaldehyde**	0.10	-	-
**Benzyl Benzoate**	0.88	-	-

[a] Taken from [Carosati E, Mattioli, L.B.; Santini A, Caprioli G, Micucci, M.; Mangiaterra G, Marzetti, C.; Scola Gagliardi M, Kan F, Scala G, Ceccarelli M, Frosini, M.; Corazza, I.; Budriesi, R. Thyme, Oregano, and Cinnamom essential oils: investigating their molecular mechanism of action for the treatment of bacteria-induced cystitis. ACS Omega, 2026, 11, 9737-9773.]
